# Photopolymerized 3D Printing Scaffolds with Pt(IV) Prodrug Initiator for Postsurgical Tumor Treatment

**DOI:** 10.34133/2022/9784510

**Published:** 2022-08-28

**Authors:** Qingfei Zhang, Xiaocheng Wang, Gaizhen Kuang, Yunru Yu, Yuanjin Zhao

**Affiliations:** ^1^Department of Rheumatology and Immunology, Nanjing Drum Tower Hospital, School of Biological Science and Medical Engineering, Southeast University, Nanjing 210096, China; ^2^Oujiang Laboratory (Zhejiang Lab for Regenerative Medicine, Vision and Brain Health), Wenzhou Institute, University of Chinese Academy of Sciences, Wenzhou 325001, China

## Abstract

Biomedical scaffolds have shown great success in postsurgical tumor treatment; their current efforts are focusing on eradicating residual tumor cells and circulating tumor cells and simultaneously repairing postoperative tissue defects. Herein, we report a novel photopolymerized 3D scaffold with Pt(IV) prodrug initiator to achieve the desired features for tumor comprehensive therapy. The Pt-GelMA scaffold was fabricated from the microfluidic 3D printing of methacrylate gelatin (GelMA) bioinks through a Pt(IV)-induced photocrosslinked process without any other additional photoinitiator and chemotherapeutic drug. Thus, the resultant scaffold displayed efficient cell killing ability against breast cancer cells in vitro and significantly inhibited the local tumor growth and distant metastases on an orthotopic postoperative breast cancer model in vivo. Besides, benefiting from their ordered porous structures and favorable biocompatibility, the scaffolds supported the cell attachment, spreading, and proliferation of normal cells in vitro; could facilitate the nutrient transportation; and induced new tissue ingrowth for repairing tissue defects caused by surgery. These properties indicate that such 3D printing scaffold is a promising candidate for efficient postoperative tumor treatment in the practical application.

## 1. Introduction

Cancer is the leading cause of death around the world, which seriously threatens human lives [1]. Surgical resection remains the preferred choice and effective way to treat malignant solid tumors in the clinic [2, 3]. However, residual cancer cells and circulating tumor cells (CTCs) after surgery will induce high incidence of local recurrence and distant metastases, which are the main factors for the high mortality of patients [4, 5]. Systemic chemotherapy is usually employed to address cancer recurrence and metastases after surgical treatment in the clinic [6]. Typically, conventional platinum(II) complexes such as cisplatin, carboplatin, and oxaliplatin are the most commonly used chemotherapies [7, 8]. Although these complexes have achieved great success for the postoperative treatment of a broad spectrum of solid tumors, the serious systemic toxicity and side effect severely limit their wide clinical application [9–11]. In addition, inevitably intraoperative loss of healthy tissues in the tumor resection area is difficult to self-heal, which seriously affects the appearance, function, and life quality of patients [12, 13]. To overcome these limitations, implant scaffold-based drug delivery systems, including films, hydrogels, and fibers, have been employed for improving chemotherapy efficacy, reducing toxicity and side effect, and repairing the healthy tissues [14–21]. Unfortunately, the postoperative chemotherapy and repair processes are hardly realized synchronously, which are commonly carried out in a separate way, resulting in low efficiency and cost. Thus, it is highly crucial to develop multifunctional implants that not only enable the eradication of residual tumor cells but also possess remarkable tissue repair capacity after surgical treatment.

**Figure 1:**
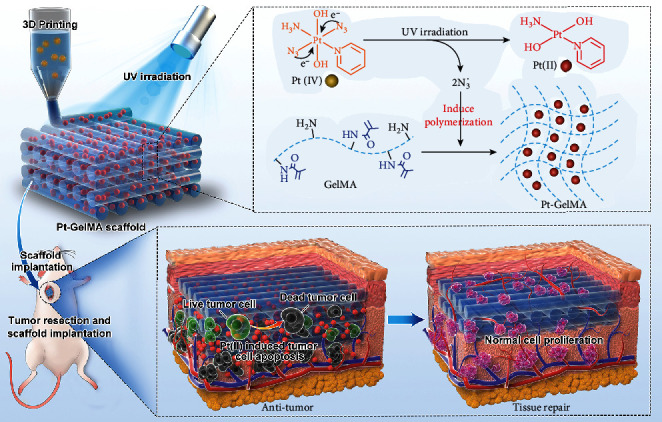
Schematic illustration of the photopolymerized three-dimensional (3D) printing scaffold with Pt(IV) prodrug initiator for postsurgical tumor treatment. Under the UV light irradiation, the Pt(IV) prodrug can be reduced to Pt(II) and generated N_3_^•^ for inducing the polymerization of GelMA bioinks, and then, the 3D Pt-GelMA scaffold could be printed by using a microfluidic 3D printing strategy. After implantation to the tumor resection site, the Pt-GelMA scaffold could efficiently kill the residual cancer cells for preventing the tumor local growth and distant metastases *in vivo*. Thereafter, after the drug release, the Pt-GelMA scaffold could serve as a 3D culture platform for the proliferation of normal cells for tissue repair.

In this paper, we present a novel photopolymerized three-dimensional (3D) printing scaffold with Pt(IV) prodrug initiator to achieve the desired multiple functions for postsurgical tumor treatment, as schemed in Figure 1. 3D printing has attracted enormous interest in the field of oncology, tissue engineering, and other medical applications in the last decades [22–24]. Multifunctional 3D-structured scaffolds with precisely controlled architectures have been widely investigated for drug delivery, cell penetration, nutrition supply, and new tissue regeneration [25–28]. Nowadays, various biopolymers such as alginate, gelatin, chitosan, and poly(lactic-co-glycolic acid) (PLGA) have been applied to fabricate 3D scaffolds for tumor therapy or tissue repair [29–35]. However, the preparation of these 3D constructs is generally initiated by additional metal ions, photopolymerization initiators, and/or other crosslinkers, which may result in a tedious preparation process and undesirable toxic side effects. In contrast, a kind of photosensitive platinum(IV) (Pt(IV)) prodrug has been extensively exploited for tumor therapy in various photoresponsive drug delivery systems, due to its transformation from low toxic Pt(IV) to high cytotoxic platinum(II) (Pt(II)) species under light irradiation [36–38]. Notably, these Pt(IV) prodrugs can generate azidyl radical (N_3_^•^) during the photoactivation process [9]. Thus, given the radicals that trigger photocrosslinking in many cases, we envision that the polymerization of Pt(IV)-prodrug-laden biocompatible scaffolds can be self-initiated by the N_3_^•^ generated from Pt(IV) prodrugs and simultaneously yields cytotoxic Pt(II) species under light irradiation, importing the scaffolds with both tumor therapeutic effects and tissue repair capacity for postsurgical tumor treatment.

To implement this innovation, we generated the Pt(IV)-prodrug-initiated photopolymerized GelMA scaffolds by employing a microfluidic 3D printing strategy and used the resultant scaffolds for preventing cancer recurrence and metastases as well as repairing the tissue defects after the surgical resection of primary tumor tissues. Microfluidic technology with high precision and controllability provided a versatile method for dealing with fluids in microchannels to prepare simple- or multicomponent microfibers [39–43]. Thus, the combination of microfluidics and 3D printing technology provides promising tactics for manufacturing 3D-structure scaffolds with high spatial and composition accuracy. Besides, as a typical platinum prodrug, *trans,trans,trans*-[Pt(N_3_)_2_(OH)_2_(NH_3_)(py)] (Pt(IV)), could act as the photopolymerization initiator to induce the photocrosslinking of GelMA scaffolds. It was found that N_3_^•^ could be efficiently generated from Pt(IV) when exposed to ultraviolet (UV) light, contributing to the polymerization of the GelMA matrix with the cytotoxic Pt(II)-loading during the microfluidic 3D printing. Based on the Pt-GelMA scaffolds, we have demonstrated that they had a significant killing effect on breast cancer 4T1 cells in vitro and also an obvious inhibition capability to the local recurrence and distant metastases on an orthotopic postoperative breast cancer model in vivo. In addition, the Pt-GelMA scaffolds with ordered porous structures and favorable biocompatibility could not only support the cell attachment, spreading, and proliferation of normal cells but also facilitate the nutrient transportation and promote new tissue ingrowth for repairing tissue defects in tumor resection sites. These fascinating features awarded the Pt(IV)-prodrug-initiated photopolymerized scaffolds with promising potential in practical postsurgical tumor treatment.

## 2. Results

**Figure 2:**
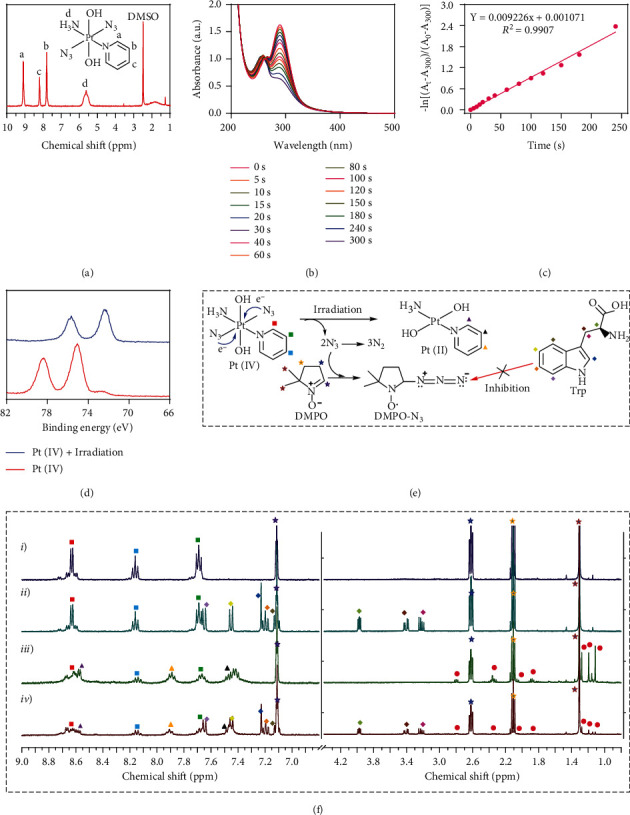
Synthesis, photoreduction, and azidyl radical (N_3_^•^) generation of Pt(IV). (a) ^1^H NMR spectrum of *trans,trans,trans*-[Pt(N_3_)_2_(OH)_2_(NH_3_)(py)] (Pt(IV)). (b) UV-vis spectra of Pt(IV) after UV irradiation for indicated time intervals. (c) The first-order kinetics of Pt(IV) degradation under UV irradiation. (d) XPS analysis of Pt(IV) before and after irradiation. (e) The schematic for the photoreduction process of Pt(IV) and the reaction of N_3_^•^ and DNPO. (f) ^1^H NMR spectra of Pt(IV) and DMPO in D_2_O: (*i*, *iii*) without Trp, (*ii*, *iv*) with Trp, (*i*, *ii*) without irradiation, and (*iii*, *iv*) irradiation for 60 s. Assignments: stars (☆): ^1^H peaks of DMPO; squares (□): ^1^H peaks of Pt(IV); triangles (△): ^1^H peaks of Pt(II); prismatic (◇): ^1^H peaks of Trp; circles (○): ^1^H peaks of DMPO-N_3_^•^ photoproducts.

Firstly, Pt(IV) was synthesized and characterized by nuclear magnetic resonance (NMR) spectroscopy and electrospray ionization mass spectrometry (ESI-MS, Figure 2(a) and Figure S1). Subsequently, the photosensitivity and azidyl radical (N_3_^•^) generation of Pt(IV) were investigated using an ultraviolet-visible-near infrared (UV-vis-NIR) spectrophotometer. The absorption peak at 289 nm was gradually decreased with a first-order kinetics after the irradiation of Pt(IV) under UV light (Figures 2(b) and 2(c)), revealing the photoreduction process of Pt(IV). This process was further detected by the X-ray photoelectron spectroscopy (XPS) measurement, which indicated that the binding energies of Pt_4f_ of Pt(IV) (78.6 and 75.1 eV) evidently changed to those of Pt(II) (75.7 and 72.5 eV) after irradiation (Figure 2(d)). As schemed in Figure 2(e), along with the Pt(IV) being reduced to Pt(II), specifically, N_3_^•^ could also be generated at the same time under the UV light irradiation. To verify this hypothesis, NMR spectroscopy was applied to investigate the generation of N_3_^•^ in the presence of dimethyl pyridine N-oxide (DMPO) as a radical spin trap. It could be found that the characteristic peak intensities of Pt(IV) (indicated as square) were significantly decreased, while the characteristic peaks of DMPO and N_3_^•^ binding product (DMPO-N_3_^•^, indicated as circle) evidently appeared with fairly high intensities after irradiation. By contrast, in the presence of tryptophan (Trp, a radical quencher), the characteristic peaks of DMPO-N_3_^•^ markedly decreased (Figure 2(f)), confirming the efficient N_3_^•^ generation of Pt(IV) under UV irradiation. Taken together, the above results indicated the successful synthesis, photoreduction, and N_3_^•^ generation of the Pt(IV) prodrug.

**Figure 3:**
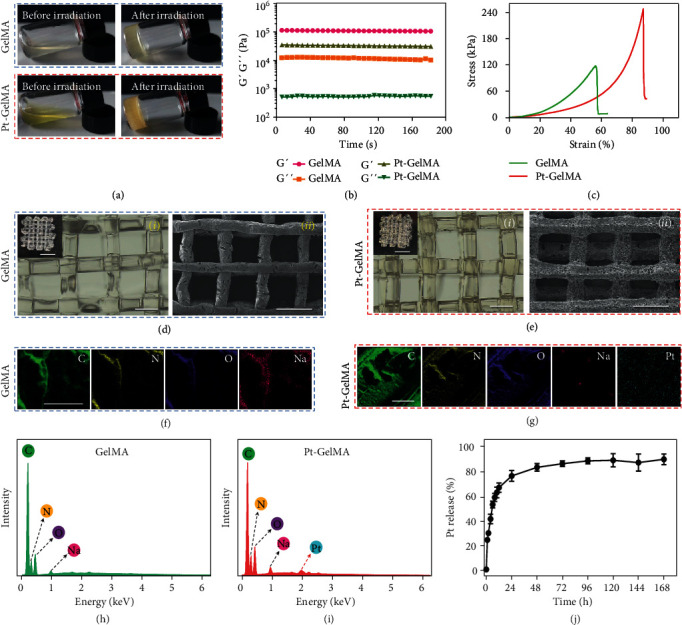
Characterization of GelMA and Pt-GelMA hydrogels, and 3D printing GelMA and Pt-GelMA scaffolds. (a) Photographs of GelMA or Pt-GelMA hydrogels at a GelMA concentration of 20% before and after UV light irradiation. (b) The storage modulus (G′) and loss modulus (G^″^) of GelMA and Pt-GelMA hydrogels. (c) The stress-strain curves of GelMA and Pt-GelMA hydrogels at a GelMA concentration of 20%. (d) Optical (*i*) and SEM image (*ii*) of GelMA scaffold. Insets in (*i*), scale bar: 5 mm; scale bar in (*i*) and (*ii*): 1000 *μ*m. (e) Optical (*i*) and SEM image (*ii*) of Pt-GelMA scaffold. Insets in (*i*), scale bar: 5 mm; scale bar in (*i*) and (*ii*): 1000 *μ*m. (f, g) Elemental mappings of GelMA (f) and Pt-GelMA (g) scaffolds. Scale bar: 50 *μ*m. (h, i) EDS spectra of GelMA (h) and Pt-GelMA (i) scaffolds. (j) The release profile of Pt from the Pt-GelMA scaffold.

As a proof of concept, the N_3_^•^-induced crosslink of GelMA with the Pt(IV) prodrug initiator was investigated. The GelMA was firstly successfully synthesized from gelatin and characterized by NMR spectroscopy as the characteristic peaks at 5.32 and 5.55 ppm of the double bond could be observed (Figure S2). Then, the GelMA solutions at different concentrations were added with a photopolymerization initiator of either Pt(IV) or lithium phenyl-2,4,6-trimethylbenzoylphosphinate (LAP). After the irradiation of UV light, the formation of GelMA or Pt-GelMA hydrogels in the transparent vials could be clearly observed (Figure 3(a) and Figure S3), indicating that the N_3_^•^ could function as a photopolymerization initiator to effectively initiate the crosslinking of GelMA. Besides, the storage modulus (G′) of GelMA and Pt-GelMA hydrogels at various GelMA concentrations was much higher than their respective loss modulus (G^″^), further confirming the formation of true gels (Figure 3(b) and Figure S4). Interestingly, a prominent 3D interconnected porous structure of Pt-GelMA hydrogels could be observed by scanning electron microscopy (SEM), which is distinct from the intact microstructure of the GelMA hydrogels photopolymerized using the LAP initiator (Figure S5). The 3D porous structure of Pt-GelMA hydrogels could be attributed to the generated nitrogen from Pt(IV) when exposed to UV light irradiation during the preparation process of Pt-GelMA hydrogels. This fascinating feature endows the Pt-GelMA hydrogels with more deformability with higher breaking compressibility than GelMA hydrogels at the same concentration; thereby, the Pt-GelMA hydrogels could be folded easily when applied for 3D printing and *in vivo* implantation (Figure 3(c) and Figure S6). To verify the universality of Pt(IV) prodrug as a photopolymerization initiator, we used the Pt(IV) to initiate the polymerization of polyethylene glycol diacrylate (PEGDA), methacrylate alginate (AlgMA), and methacrylate hyaluronic acid (HAMA) solutions. It was found that the formation of PEGDA, AlgMA, and HAMA hydrogels could be clearly observed in the transparent vials, greatly similar to the GelMA, indicating the universality of Pt(IV) to initiate photopolymerization of multiple polymers (Figure S7).

Subsequently, the capacity of 3D printing of the Pt(IV) and GelMA mixture was investigated in detail. As shown in Figure S8, the microfluidic chip was applied as a 3D printing head to be integrated into a programmable 3D printer. The fluid of the GelMA solution containing LAP could be firstly extruded from the capillary microfluidic device to generate continuous microfibers after UV light irradiation, and then, the microfibers could be stacked to form a 3D scaffold with the programmable 3D printer. As shown in Figure S9, the 3D printing GelMA scaffolds were successfully prepared with various shapes and sizes such as triangle, circle, and square. Similarly, the Pt(IV)-induced photocrosslinked GelMA 3D scaffold (Pt-GelMA) was also printed (Figure S10). It was found that both GelMA and Pt-GelMA scaffolds had well-organized and regular 3D architectures under the observation of an optical microscope and SEM (Figures 3(d) and 3(e)). Different from the GelMA scaffold, the Pt element was uniformly distributed in energy dispersion spectroscopy (EDS) elemental mappings of the Pt-GelMA scaffold (Figures 3(f)–3(i)), indicating the effective drug loading of the 3D scaffold. Subsequently, the drug release behavior of the Pt-GelMA scaffold was investigated, and the result showed that about 75% of the loaded Pt could be released within 24 h (Figure 3(j)), which is favorable for the further application of postsurgical tumor chemotherapy. The rapid release of drugs at a high concentration can effectively kill the residual cancer cells, and then, the 3D scaffold with negligible remaining drugs could further provide a bioactive platform for the migration, adhesion, and ingrowth of normal cells.

**Figure 4:**
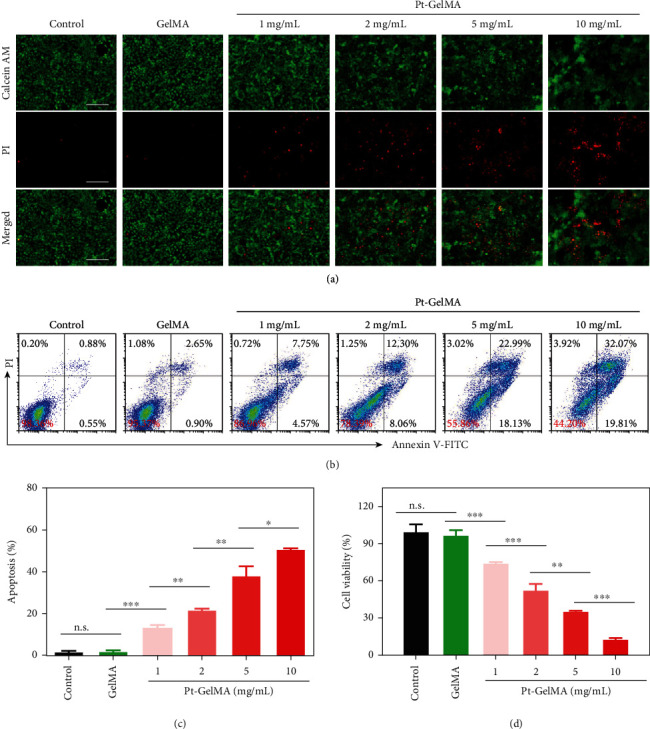
*In vitro* cytotoxicity assay of Pt-GelMA scaffold against cancer cells. (a) Live/dead staining of 4T1 cells after being incubated with Pt-GelMA scaffolds with different drug loading concentrations for 24 h. Scale bar: 100 *μ*m. (b) Apoptosis analysis and (c) corresponding apoptosis rates of 4T1 cells after incubation with different scaffolds for 24 h by flow cytometry. (d) CCK8 assay of 4T1 cells after being incubated with different scaffolds for 24 h. All the cell experiments had three independent replicates (*n* = 3). n.s.: no significance. ^∗^*p* < 0.05, ^∗∗^*p* < 0.01, ^∗∗∗^*p* < 0.001.

Having confirmed the successful fabrication of the Pt-GelMA scaffold with the Pt(IV) prodrug initiator, the *in vitro* inhibition efficacy of the scaffold against cancer cells (breast tumor 4T1 cells as model cells) was further investigated using Calcein-AM/propidium iodide (PI) staining. As shown in Figure 4(a), the 4T1 cells were significantly killed when incubating with the Pt-GelMA scaffold, and the cytotoxicity effect was evidently increased with the increasing drug concentrations (Figure 4(a)). In remarkable contrast, the GelMA scaffold without drug loading had a minimal influence on the proliferation of the 4T1 cells. Quantitatively, the apoptosis rates of 4T1 cells of the control and GelMA scaffold treatment groups were 1.91% and 2.12% by flow cytometry, respectively, which were significantly lower than those of Pt-GelMA scaffold treatment groups. The apoptosis rates were 13.33% (1 mg mL^−1^), 21.83% (2 mg mL^−1^), 38.26% (5 mg mL^−1^), and 50.81% (10 mg mL^−1^), respectively (Figures 4(b) and 4(c)). Cell counting kit-8 (CCK8) assay further confirmed the cell killing effects of the Pt-GelMA scaffold with a substantially decreased viability of 4T1 cells in Pt-GelMA groups (Figure 4(d)), indicating the excellent tumor therapeutic efficiency of Pt-GelMA scaffold *in vitro*.

**Figure 5:**
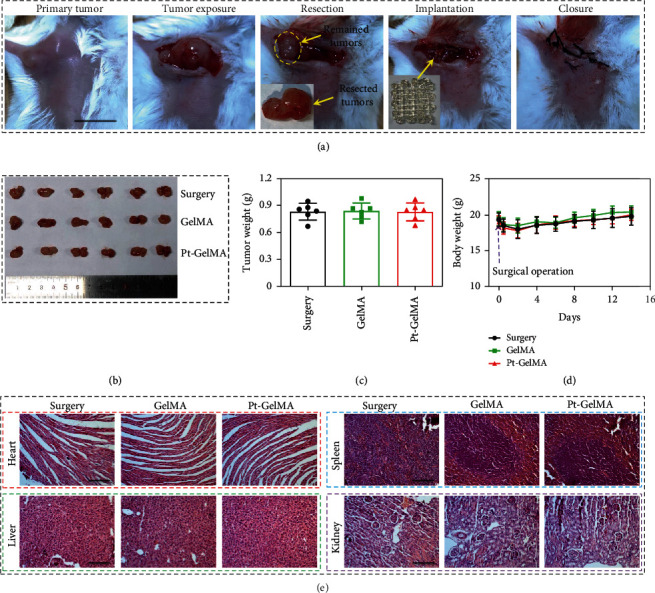
*In vivo* antitumor procedure and biosafety of the Pt-GelMA scaffold. (a) The tumor resection and scaffold implantation processes. Scale bar: 1 cm. (b) Photograph and (c) weight of the resected tumors. (d) The changes of body weight of different mice during the experiment. (e) H&E staining of heart, liver, spleen, and kidney of the mice after different treatments. Scale bar: 100 *μ*m. Each experiment group has 6 mice (*n* = 6).

To investigate the tumor eradication capacity and tissue repair ability of Pt-GelMA scaffold for postsurgical tumor treatment, an incomplete resection orthotopic breast tumor mice model was established *in vivo*. As illustrated in Figure 5(a), 90% of tumor tissues were resected, and the remaining tumor volume was ~30 mm^3^. The photographs and weights of the resection tumors are shown in Figures 5(b) and 5(c), and the mice were then accordingly divided into surgery, GelMA, and Pt-GelMA treatment groups; it demonstrated that the sectionalization is even. Subsequently, a GelMA or Pt-GelMA scaffold was implanted into the tissue defects after tumor resection and sutured (Figure 5(a)). The mice without scaffold implantation were set as the control. After the different treatments, the mice of each group were weighted every two days, and the body weights were recorded, which were gradually increased from day 2 (Figure 5(d)). Besides, no obvious pathological damage was found in H&E staining of major organs from each group, indicating the excellent *in vivo* biosafety of the 3D scaffolds (Figure 5(e)).

**Figure 6:**
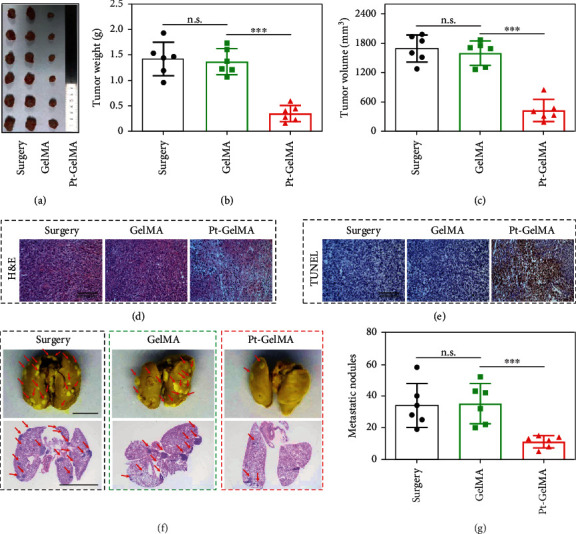
*In vivo* tumor inhibition and antimetastasis ability of the Pt-GelMA scaffold. (a) Photograph, (b) tumor weights, and (c) tumor volumes of 4T1-tumor-bearing mice after different treatments. (d) H&E and (e) TUNEL analyses of different groups. Scale bar: 100 *μ*m. (f) Representative photographs and H&E staining of lungs with pulmonary metastatic nodules (indicated with red arrow) after different treatments. Scale bar: 5 mm. (g) Quantification of the pulmonary metastatic nodules after different treatments. Each experiment group has 6 mice (*n* = 6). n.s.: no significance. ^∗∗∗^*p* < 0.001.

Then, the tumor inhibition and antimetastasis ability of the Pt-GelMA scaffold was further evaluated two weeks after the scaffold implantation. It could be found that the Pt-GelMA scaffolds substantially inhibited the tumor growth, showing the smallest tumor weight and volume, as compared to the uncontrolled growth of tumors in the surgery and GelMA groups (Figures 6(a)–6(c)). Besides, hematoxylin and eosin (H&E) and terminal-deoxynucleoitidyl transferase mediated nick end labeling (TUNEL) staining of the tumor sections indicated that the treatment of Pt-GelMA scaffolds led to the most extensive cellular destruction and apoptosis in comparison with the other two groups (Figures 6(d) and 6(e)). Furthermore, the tumor metastases to lungs were observed from the photographs and H&E staining (Figure 6(f)). The results showed that the Pt-GelMA group had the least metastatic tumor nodules (≈11 per lung) compared with the surgery (≈34 per lung) and GelMA (≈35 per lung) groups (Figure 6(g)), suggesting the evident antimetastasis ability of the Pt-GelMA scaffold.

**Figure 7:**
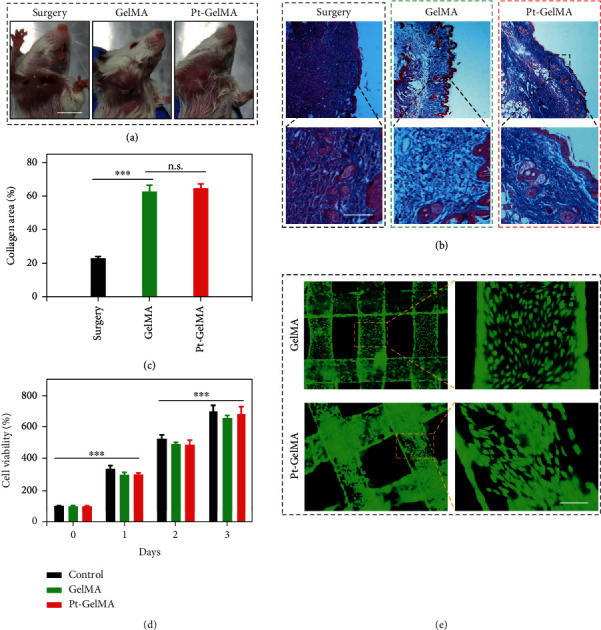
Tissue regenerative ability of the Pt-GelMA scaffold. (a) The photographs of tumor-bearing mice two weeks after different treatments. Scale bar: 1 cm. (b) Representative Masson's trichrome staining images of the skin tissues on day 14. Scale bar: 50 *μ*m. (c) Quantification of collagen deposition in different groups. (d) Cell activity of NIH 3T3 cells cultured with different scaffolds. (e) Live/dead staining images of NIH 3T3 cells cultured on the scaffolds. Scale bar: 200 *μ*m. Each experiment group has 6 mice (*n* = 6). n.s.: no significance. ^∗∗∗^*p* < 0.001.

For the treatment of postoperative tumors, in addition to antitumor and antimetastases, the tissue repair is of great importance for the health of patients. To our delight, the GelMA and Pt-GelMA scaffolds were found to promote the wound healing at the surgical sites at the end of experiments (Figure 7(a)). Masson's trichrome staining further revealed the enhanced collagen deposition in the groups treated with GelMA and Pt-GelMA scaffolds as compared to the control group (Figures 7(b) and 7(c)), indicating the tissue regenerative ability of the scaffolds. Therefore, we explored the in vitro bioactivity of the GelMA and Pt-GelMA scaffolds by incubation with normal cells. Before culturing normal cells, the drugs in the Pt-GelMA scaffold were firstly released. Then, both the GelMA and Pt-GelMA scaffolds without drug loading were used to evaluate the 3D cell culture ability. The CCK8 assay demonstrated that the cell viabilities of mouse normal fibroblasts (NIH 3T3 cells) significantly increased with the extension of culture time (Figure 7(d)). Furthermore, the 3D scaffolds could efficiently support the attachment, expansion, and ingrowth of normal cells (Figure 7(e)), indicating the excellent *in vitro* biocompatibility of the Pt-GelMA scaffold.

## 3. Discussion

In summary, we have reported a 3D printing Pt(IV)-prodrug-induced photopolymerized GelMA scaffold for cancer postsurgical therapy. The 3D Pt-GelMA scaffold was fabricated from GelMA solution containing Pt(IV) prodrugs by using a microfluidic printing strategy. Here, the synthesized Pt(IV) could not only be photoreduced to Pt(II) for efficient tumor cell killing but also induce the generation of N_3_^•^ for GelMA crosslinking. Therefore, the obtained Pt-GelMA scaffold could significantly suppress the proliferation of tumor cells *in vitro* and pronouncedly prevented the tumor local growth and distant metastases of tumors *in vivo* without noticeable system toxicity. Moreover, after the drug release, the Pt-GelMA scaffold could serve as a 3D culture platform for normal cell adhesion and proliferation, which is helpful for healthy tissue regeneration after surgery. Taken together, these fascinating characters render the Pt-GelMA scaffold as a great prospect in the practical clinical treatment of postoperative tumors.

## 4. Materials and Methods

### 4.1. Materials

Cisplatin was purchased from Platinum Energy Co. Ltd., China. Silver nitrate (AgNO_3_), dimethyl pyridine N-oxide (DMPO), pyridine (py), tryptophan (Trp), sodium azide (NaN_3_), and hydrogen peroxide solution (H_2_O_2_) were purchased from Aladdin.

### 4.2. Synthesis of *trans,trans,trans*-[Pt(N_3_)_2_(OH)_2_(NH_3_)(py)] (Pt(IV))

In brief, 300 mg of cisplatin was suspended in 20 mL of H_2_O and added with pyridine (py) (790 mg) for stirring for 2 h at 75°C. Thereafter, the solution was evaporated to get a white powder, followed by adding HCl solution (2 M, 3 mL) and stirring at 75°C for 3 days to obtain a yellow solid. The solid was then filtered to obtain *trans*-[PtCl_2_(NH_3_)(py)]. Subsequently, 362 mg of *trans*-[PtCl_2_(NH_3_)(py)] was suspended in 20 mL of H_2_O and added with AgNO_3_ (0.34 g, 2 mmol) for stirring for 24 h. Then, NaN_3_ (130 mg, 2 mmol) was added, followed by stirring for another 6 h to get a yellow precipitate. The yellow solid was filtered and washed before drying to get *trans*-[Pt(N_3_)_2_(NH_3_)(py)]. Finally, H_2_O_2_ (30%) was used to oxidize *trans*-[Pt(N_3_)_2_(NH_3_)(py)] to get the Pt(IV). ^1^H NMR (ppm): 9.09, 8.19, 7.80, and 5.63. ^13^C NMR (ppm): 147.60, 141.68, and 126.24. ESI-MS (m/z): [M+Na]^+^ 432.1.

### 4.3. Photoreduction of Pt(IV)

The photoreduction behavior of Pt(IV) was detected by UV-Vis spectrometer (CARY 5000, USA). The solution of Pt(IV) (20 *μ*g/mL) was irradiated by UV light (365 nm, 1.25 W/cm^2^) for preset time points before being detected by UV-Vis spectra. After photoreduction, the solution was lyophilized for XPS measurement.

### 4.4. Detection of Azidyl Radical (N_3_^•^)

The N_3_^•^ generation from Pt(IV) was detected by using ^1^H-NMR spectroscopy (Bruker AVANCE DRX 400, Germany). Briefly, Pt(IV) dissolved in D_2_O (10 mM) was added to the DMPO solution (20 mM) with or without Trp (2 mM). Thereafter, the reaction solutions were treated with two ways: the one without irradiation and the one irradiated by UV light for 1 min. At last, all these solutions were investigated by ^1^H-NMR spectroscopy.

### 4.5. Synthesis of GelMA

In a typical experiment process, gelatin (20 g) was added to a solution of 200 mL H_2_O containing Na_2_CO_3_ (10 g) and stirred for 2 h. Thereafter, methacrylic anhydride (5 mL) was dropwise added within 30 min, and the solution was stirred for another 2 h. During the reaction process, the pH value of the solution was maintained at 8-9, which was adjusted by NaOH solution (1 M). After that, the solution was dialyzed against deionized water before lyophilization to obtain GelMA.

### 4.6. The Formation of GelMA Hydrogel and Pt-GelMA Hydrogel

For the formation of GelMA hydrogels, pregel solutions composed of GelMA with different concentrations (10%, 20%, and 30%) and LAP (0.1% *w*/*v*) were prepared. Then, the pregel solutions were irradiated by UV light for 60 s. For the formation of Pt-GelMA hydrogels, pregel solutions composed of GelMA with different concentrations (10%, 20%, and 30%) and Pt(IV) (0.1% *w*/*v*) were prepared. These pregel solutions were also irradiated by UV light for 60 s. The mechanical performance of the obtained GelMA hydrogels and Pt-GelMA hydrogels such as compressive stress-strain, storage modulus (G′), and loss modulus (G^″^) was investigated. In addition, the Pt(IV) initiated the polymerization of polyethylene glycol diacrylate (PEGDA, 10%), methacrylate alginate (AlgMA, 2.5%), and methacrylate hyaluronic acid (HAMA, 5%) which was also investigated using the same method.

### 4.7. Microfluidic 3D Printing Drug-Induced Photocrosslinked GelMA Scaffold

For the fabrication of the 3D printing scaffold, a pregel solution composed of Pt(IV) (0.1% *w*/*v*) and GelMA (20%) was prepared. Then, the Pt(IV)-doped GelMA solution was transfused into a capillary microfluidic device for 3D printing. The flow rate of the Pt(IV)-doped GelMA solution was set at 3 mL h^−1^, and the moving speed of the 3D printer was set at 5 mm s^−1^. During the printing process, the 3D printing Pt-GelMA scaffold was obtained in the PBS solution under the exposure to UV light irradiation. The GelMA scaffolds without Pt(IV) were prepared as the control under the same experimental conditions.

### 4.8. Characterizations

Optical photographs of the scaffolds were observed with a stereomicroscope (Olympus BX51, Tokyo, Japan). The morphology, structure, and surface element analysis of the scaffolds were investigated using a field emission scanning electron microscope (SEM, SU8010, Hitachi, Japan). To maintain the microstructure of the hydrogels, the hydrogels were dehydrated in a series of 70%, 80%, 90%, and 100% ethanol and then dried using a supercritical drying method before observation under SEM.

### 4.9. Drug Release *In Vitro*

The release behavior of Pt from the Pt-GelMA scaffold was investigated. Briefly, the Pt-GelMA scaffolds were immersed into a 20 mL PBS solution and maintained in a shaking culture incubator (100 rpm) at 37°C for 168 h. After being incubated with the indicated time, the supernatant was extracted and added with fresh release medium (1 mL). Finally, the Pt content in the supernatant was detected and recorded by inductively coupled plasma mass spectrometry (ICP-MS).

### 4.10. *In Vitro* Cytotoxicity Assay of Drug-Loaded 3D Scaffolds against Cancer Cells

2.5 × 10^5^ cells/well of mouse breast cancer 4T1 cells were seeded in a 12-well plate and incubated overnight for cell attachment. After that, 1 mg/mL, 2 mg/mL, 5 mg/mL, and 10 mg/mL Pt-GelMA 3D scaffolds with Pt-drug contents of 62.5 *μ*g, 125 *μ*g, 312.5 *μ*g, and 625 *μ*g, respectively, were put into each well and coincubated for 24 h. The cytotoxicity of drug-loaded 3D scaffolds against cancer cells was examined using Live/Dead staining assay, CCK8 assay, and annexin V/PI apoptosis detection. For the Live/Dead staining study, cells of each well were stained by Calcein-AM/PI (Thermo Fisher Scientific, USA) and observed under a fluorescence microscope. For the CCK8 measurement, the cells were added with CCK8 reagent and cultured for another 2 h before being measured by a microplate reader. For apoptosis detection, the cells were digested and collected, followed by costaining with annexin V and PI. Then, the apoptosis of each group was detected by flow cytometry.

### 4.11. *In Vivo* Antitumor Ability of the Pt-GelMA 3D Scaffold

BALB/c mice (female, 16-18 g) were provided by Changzhou Kavins Laboratory Animal Co., Ltd. Animal experiments were performed under the guidance and approval of the Laboratory Animal Care and Use Ethical Committee of Oujiang Laboratory (Zhejiang Lab for Regenerative Medicine, Vision and Brain Health), Wenzhou Institute, University of Chinese Academy of Sciences. To establish the orthotopic breast tumor model, 4T1 cells (4 × 10^6^ cells per mouse) were injected into the right mammary, and the tumor sizes were allowed to increase to 300 mm^3^. To simulate the postsurgical residual tumors, 90% of the tumor of each mouse was surgically removed, and the retained tumor volume was about 30 mm^3^. Thereafter, the mice received different treatments: surgery, GelMA (implanted with GelMA scaffolds), and Pt-GelMA (implanted with Pt-GelMA scaffolds at a Pt concentration of 250 *μ*g per mouse). After the experiment, the weight of the mice was recorded every other day. At day 14, the mice were euthanized, and the tumors, spleen, kidney heart, liver, and skin tissues around the tumor sites were harvested and fixed in 4% (*v*/*v*) paraformaldehyde. The lungs were immersed into Bouin's solution for the observation of tumor metastatic nodules. Subsequently, cut the tumors and organs into 5 *μ*m thick sections before staining with H&E. The skin tissue sections were also stained with Masson's trichrome.

### 4.12. *In Vitro* Biocompatibility Study of 3D Scaffolds

The *in vitro* biocompatibility of the 3D scaffolds was evaluated by culturing with the mouse fibroblasts (NIH 3T3 cells). The drug in Pt-GelMA scaffolds was firstly released before culturing with NIH 3T3 cells. Then, the GelMA and Pt-GelMA scaffolds without drug loading were put in a 12-well plate. Subsequently, the 3T3 cells (2.5 × 10^5^ cells/well) were seeded on the scaffolds and cultured for different time intervals. For the cell proliferation test, the cells were cultured with scaffolds for 0, 1, 2, and 3 days, and their viabilities were evaluated by the CCK8 assay. For cell adhesion observation, the cells were cultured with scaffolds for 2 days, costained with Calcein-AM/PI, followed by visualizing via a fluorescence microscope.

### 4.13. Statistical Analysis

*In vivo* antitumor experiments had six replicates (*n* = 6). Unless otherwise specified, the other experiments had triple replicates (*n* = 3). Data are presented as the mean ± SD. Statistical significance was calculated via unpaired Student's *t*-tests. ^∗^*p* < 0.05, ^∗∗^*p* < 0.01, and ^∗∗∗^*p* < 0.001.

## Data Availability

All data used to support the findings in the paper and supplementary materials are available from the corresponding author upon reasonable request.

## Supplementary Materials

Supplementary Materials Figure S1: (a) synthesis rout and chemical structure of Pt(IV). (b) ^1^H NMR spectra of *trans*-[PtCl_2_(NH_3_)(py)] (b) and *trans*-[Pt(N_3_)_2_(NH_3_)(py)] (c) in d_6_-DMSO. (d) ^13^C NMR spectrum of Pt(IV) in d_6_-DMSO. (e) ESI-MS of Pt(IV). Figure S2: ^1^H NMR spectra of gelatin and GelMA in D_2_O. Figure S3: photographs of (a, b) GelMA and (c, d) Pt-GelMA hydrogels with the GelMA concentrations of 10% and 30% before and after UV light irradiation. Figure S4: (a, b) the storage modulus (G′) and loss modulus (G^″^) of GelMA hydrogels at GelMA concentrations of 10% (a) and 30% (b). (c, d) The storage modulus (G′) and loss modulus (G^″^) of Pt-GelMA hydrogels at GelMA concentrations of 10% (c) and 30% (d). Figure S5: (a, b) photographs of GelMA (a) and Pt-GelMA (b) hydrogels at GelMA concentrations of 10%, 20%, and 30%. Scale bare: 1 cm. (c, d) SEM images of GelMA (c) and Pt-GelMA (d) hydrogels at a GelMA concentration of 20%. Figure S6: (a, b) the stress-strain curves of GelMA hydrogels at a GelMA concentration of 10% (a) and 30% (b). (c, d) The stress-strain curves of Pt-GelMA hydrogels at a GelMA concentration of 10% (c) and 30% (d). Figure S7: photographs of (a) PEGDA, (b) AlgMA, and (c) HAMA hydrogels with the concentrations of 10%, 2.5% and 5%, respectively, before and after UV light irradiation. Figure S8: photograph of the microfluidic 3D printer with capillary microfluidic chips for scaffold printing. Scale bar, 1000 *μ*m. Figure S9: photograph of representative 3D printing GelMA scaffolds with designed sizes and shapes. Figure S10: photograph of the 3D printing Pt-GelMA scaffold.
