# Supplementary material for: Photopolymerized 3D Printing Scaffolds with Pt(IV) Prodrug Initiator for Postsurgical Tumor Treatment
Source: Research (Wash D C). 2022 Aug 28;2022:9784510. doi: 10.34133/2022/9784510 (PMC9448443; doi:10.34133/2022/9784510)
Supplement: Supplementary Materials — Figure S1: (a) synthesis rout and chemical structure of Pt(IV). (b) 1H NMR spectra of trans-[PtCl2(NH3)(py)] (b) and trans-[Pt(N3)2(NH3)(py)] (c) in d6-DMSO. (d) 13C NMR spectrum of Pt(IV) in d6-DMSO. (e) ESI-MS of Pt(IV). Figure S2: 1H NMR spectra of gelatin and GelMA in D2O. Figure S3: photographs of (a, b) GelMA and (c, d) Pt-GelMA hydrogels with the GelMA concentrations of 10% and 30% before and after UV light irradiation. Figure S4: (a, b) the storage modulus (G′) and loss modulus (G″) of GelMA hydrogels at GelMA concentrations of 10% (a) and 30% (b). (c, d) The storage modulus (G′) and loss modulus (G″) of Pt-GelMA hydrogels at GelMA concentrations of 10% (c) and 30% (d). Figure S5: (a, b) photographs of GelMA (a) and Pt-GelMA (b) hydrogels at GelMA concentrations of 10%, 20%, and 30%. Scale bare: 1 cm. (c, d) SEM images of GelMA (c) and Pt-GelMA (d) hydrogels at a GelMA concentration of 20%. Figure S6: (a, b) the stress-strain curves of GelMA hydrogels at a GelMA concentration of 10% (a) and 30% (b). (c, d) The stress-strain curves of Pt-GelMA hydrogels at a GelMA concentration of 10% (c) and 30% (d). Figure S7: photographs of (a) PEGDA, (b) AlgMA, and (c) HAMA hydrogels with the concentrations of 10%, 2.5% and 5%, respectively, before and after UV light irradiation. Figure S8: photograph of the microfluidic 3D printer with capillary microfluidic chips for scaffold printing. Scale bar, 1000 μm. Figure S9: photograph of representative 3D printing GelMA scaffolds with designed sizes and shapes. Figure S10: photograph of the 3D printing Pt-GelMA scaffold. [file 9784510.f1.doc]

Title

Photo-polymerized 3D printing scaffolds with Pt(IV) prodrug initiator for post-surgical tumor treatment

Pt(IV)-initiated photo-polymerized 3D printing scaffolds

**Authors**

Qingfei Zhang^1,2^, Xiaocheng Wang^1,2^, Gaizhen Kuang^1,2^, Yunru Yu^1,2^, Yuanjin Zhao^1,2^*

**Affiliations**

^1^ Department of Rheumatology and Immunology, Nanjing Drum Tower Hospital, School of Biological Science and Medical Engineering, Southeast University, Nanjing 210096, China

^2^ Oujiang Laboratory (Zhejiang Lab for Regenerative Medicine, Vision and Brain Health), Wenzhou Institute, University of Chinese Academy of Sciences, Wenzhou 325001, China

*Corresponding author: Yuanjin Zhao, yjzhao@seu.edu.cn

**Supplementary Materials**

Figure S1. (a) Synthesis rout and chemical structure of Pt(IV). (b) ^1^H NMR spectra of *trans*-[PtCl_2_(NH_3_)(py)] (b) and *trans*-[Pt(N_3_)_2_(NH_3_)(py)] (c) in d_6_-DMSO. (d) ^13^C NMR spectrum of Pt(IV) in d_6_-DMSO. (e) ESI-MS of Pt(IV). Figure S2. ^1^H NMR spectra of gelatin and GelMA in D_2_O. Figure S3. Photographs of (a, b) GelMA and (c, d) Pt-GelMA hydrogels with the GelMA concentrations of 10% and 30% before and after UV light irradiation. Figure S4. (a, b) The storage modulus (G′) and loss modulus (G″) of GelMA hydrogels at GelMA concentrations of 10% (a) and 30% (b). (c, d) The storage modulus (G′) and loss modulus (G″) of Pt-GelMA hydrogels at GelMA concentrations of 10% (c) and 30% (d). Figure S5. (a, b) Photographs of GelMA (a) and Pt-GelMA (b) hydrogels at GelMA concentrations of 10%, 20%, and 30%. Scale bare: 1 cm. (c, d) SEM images of GelMA (c) and Pt-GelMA (d) hydrogels at a GelMA concentration of 20%. Figure S6. (a, b) The stress-strain curves of GelMA hydrogels at a GelMA concentration of 10% (a) and 30% (b). (c, d) The stress-strain curves of Pt-GelMA hydrogels at a GelMA concentration of 10% (c) and 30% (d). Figure S7. Photographs of (a) PEGDA, (b) AlgMA and (c) HAMA hydrogels with the concentrations of 10%, 2.5% and 5% respectively before and after UV light irradiation. Figure S8. Photograph of the microfluidic 3D printer with capillary microfluidic chips for scaffold printing. Scale bar, 1000 μm. Figure S9. Photograph of representative 3D printing GelMA scaffolds with designed sizes and shapes. Figure S10. Photograph of the 3D printing Pt-GelMA scaffold.


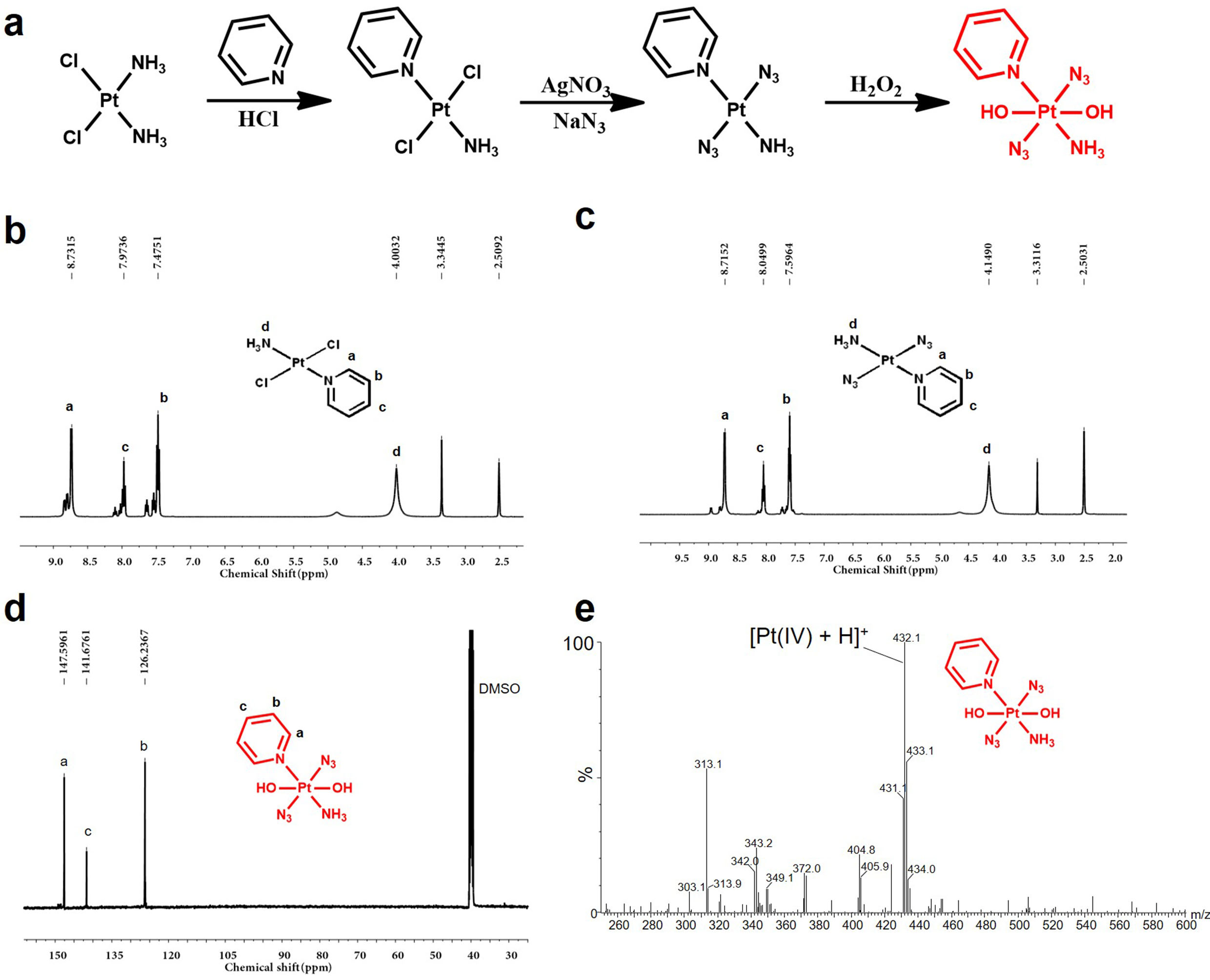


**Figure S1.** (a) Synthesis rout and chemical structure of Pt(IV). (b) ^1^H NMR spectra of *trans*-[PtCl_2_(NH_3_)(py)] (b) and *trans*-[Pt(N_3_)_2_(NH_3_)(py)] (c) in d_6_-DMSO. (d) ^13^C NMR spectrum of Pt(IV) in d_6_-DMSO. (e) ESI-MS of Pt(IV).


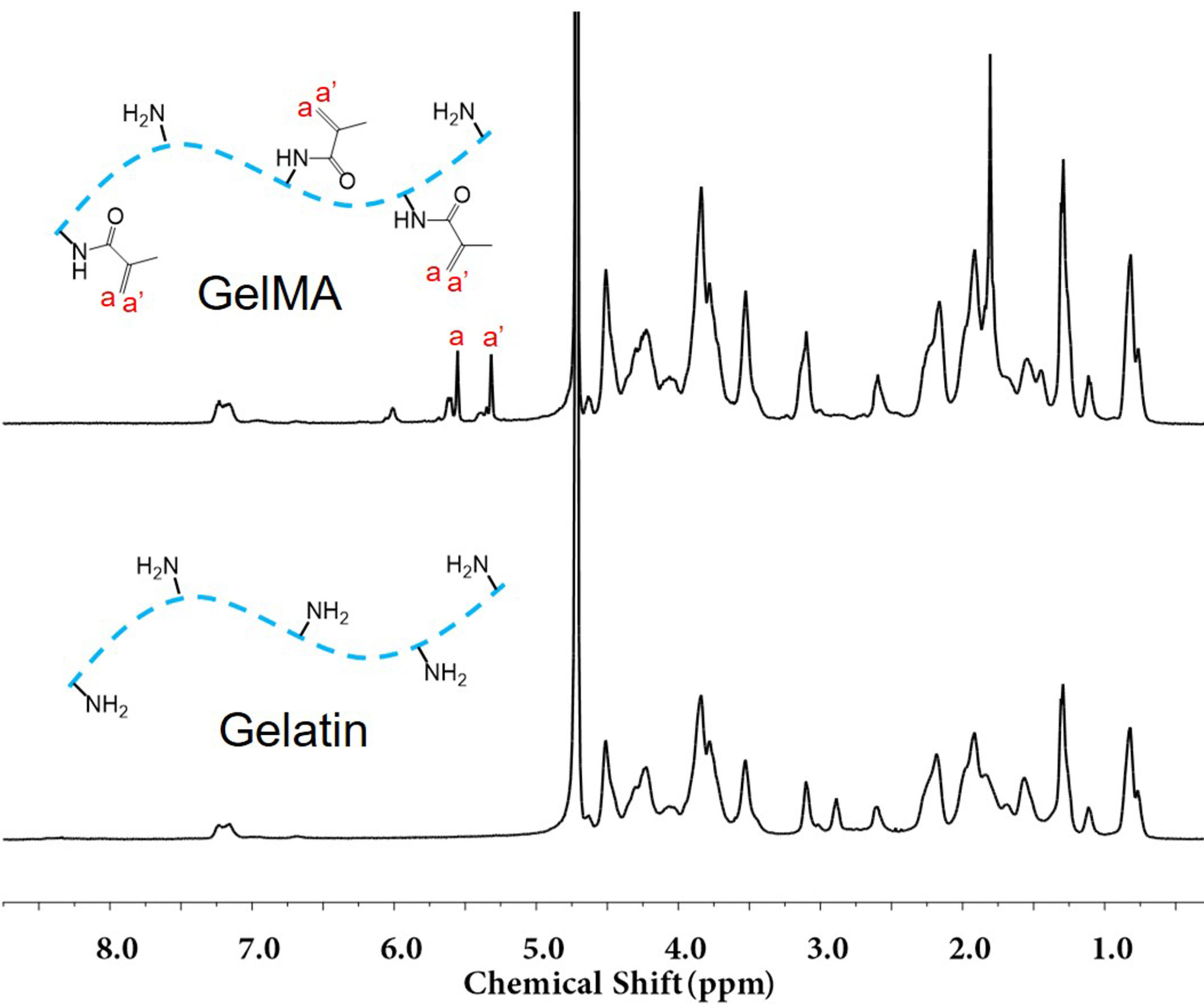


**Figure S2.** ^1^H NMR spectra of gelatin and GelMA in D_2_O.


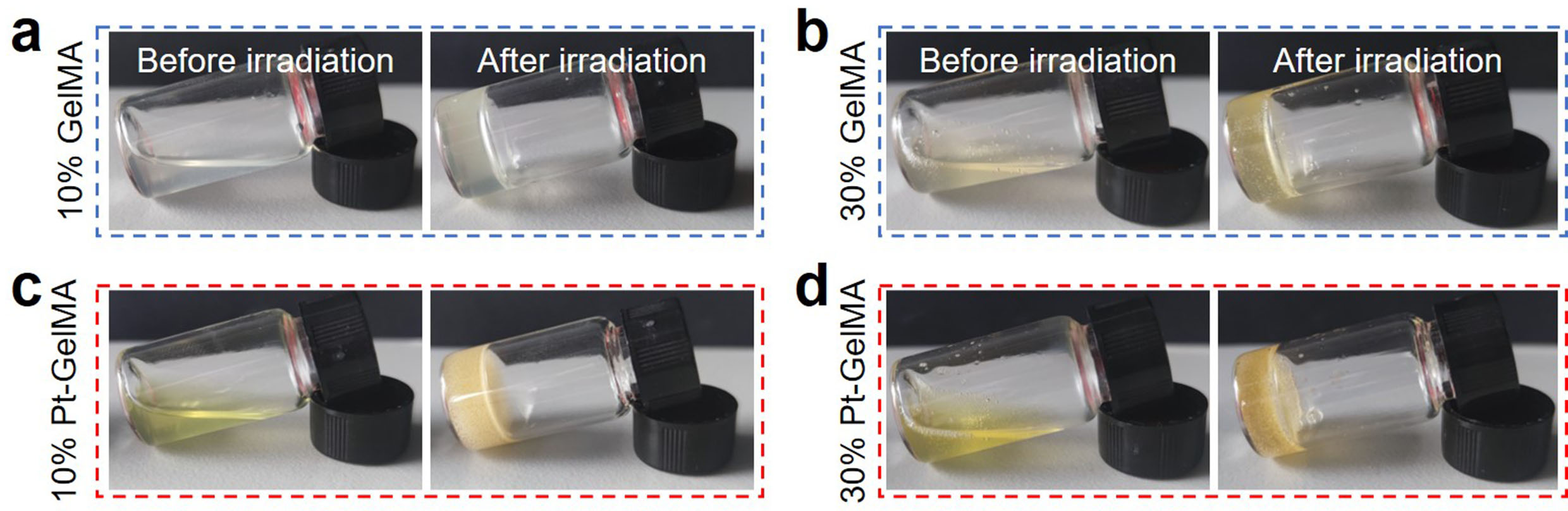


**Figure S3.** Photographs of (a, b) GelMA and (c, d) Pt-GelMA hydrogels with the GelMA concentrations of 10% and 30% before and after UV light irradiation.


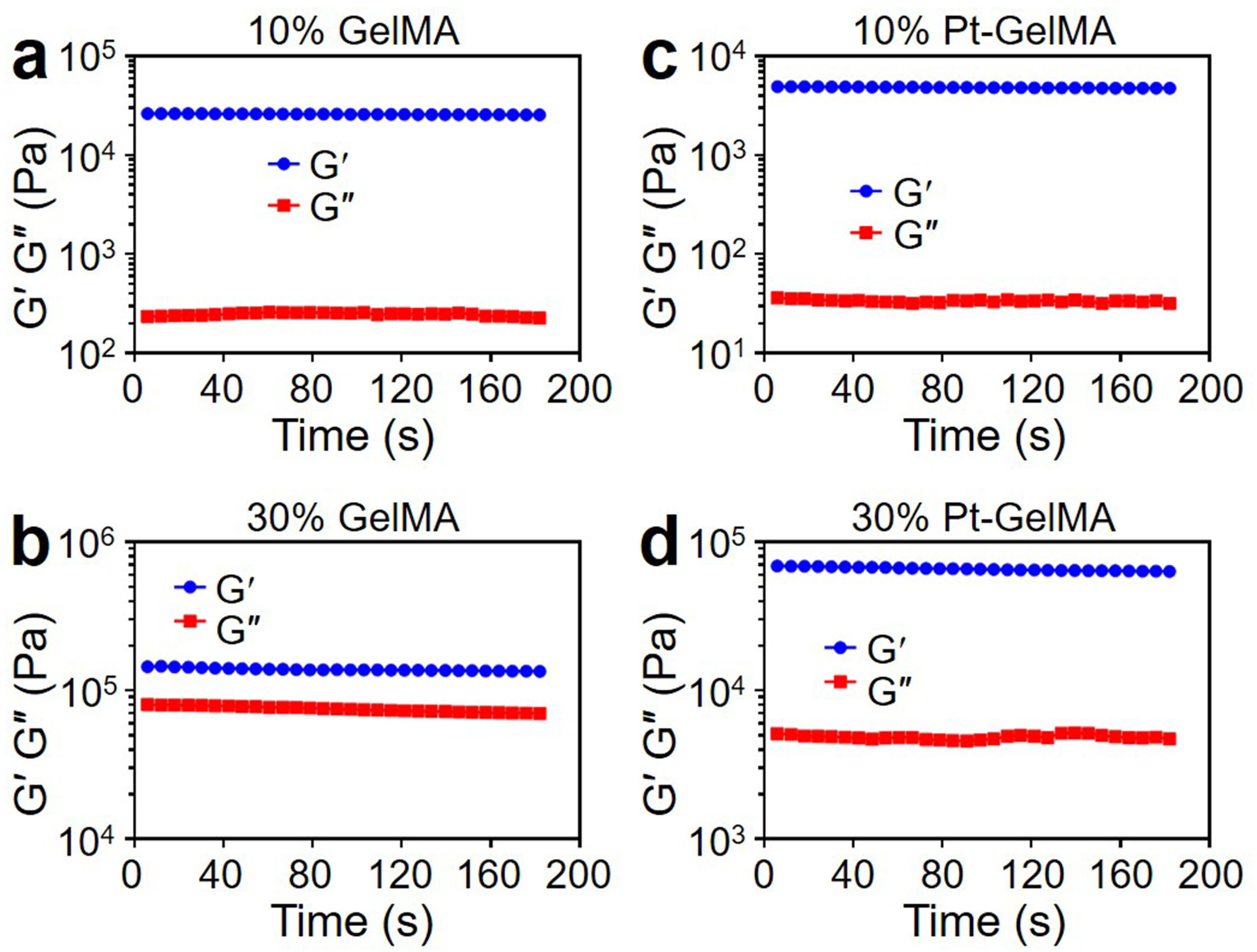


**Figure S4.** (a, b) The storage modulus (G′) and loss modulus (G″) of GelMA hydrogels at a GelMA concentration of 10% (a) and 30% (b). (c, d) The storage modulus (G′) and loss modulus (G″) of Pt-GelMA hydrogels at GelMA concentrations of 10% (c) and 30% (d).


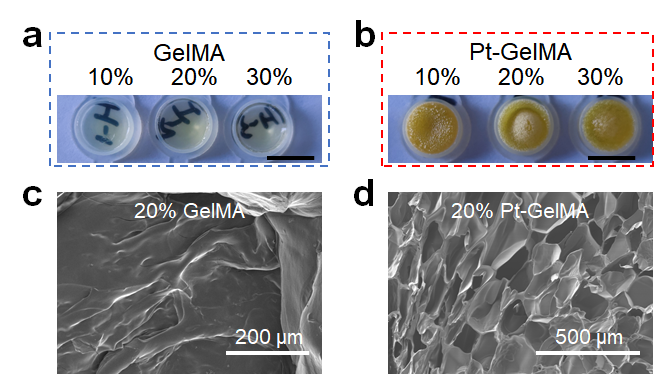


**Figure S5.** (a, b) Photographs of GelMA (a) and Pt-GelMA (b) hydrogels at GelMA concentrations of 10%, 20%, and 30%. Scale bare: 1 cm. (c, d) SEM images of GelMA (c) and Pt-GelMA (d) hydrogels at a GelMA concentration of 20%.


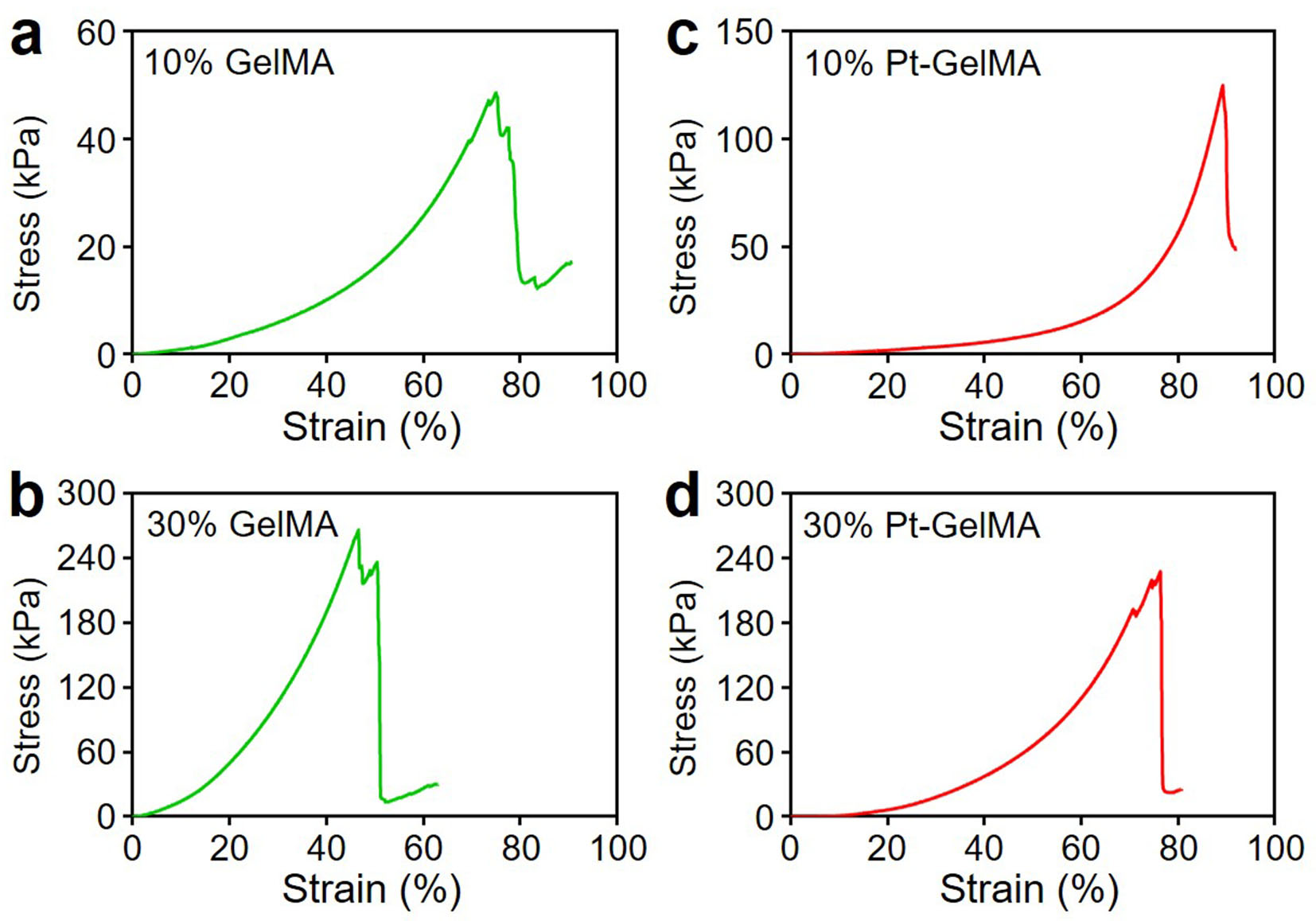


**Figure S6.** (a, b) The stress-strain curves of GelMA hydrogels at a GelMA concentration of 10% (a) and 30% (b). (c, d) The stress-strain curves of Pt-GelMA hydrogels at a GelMA concentration of 10% (c) and 30% (d).


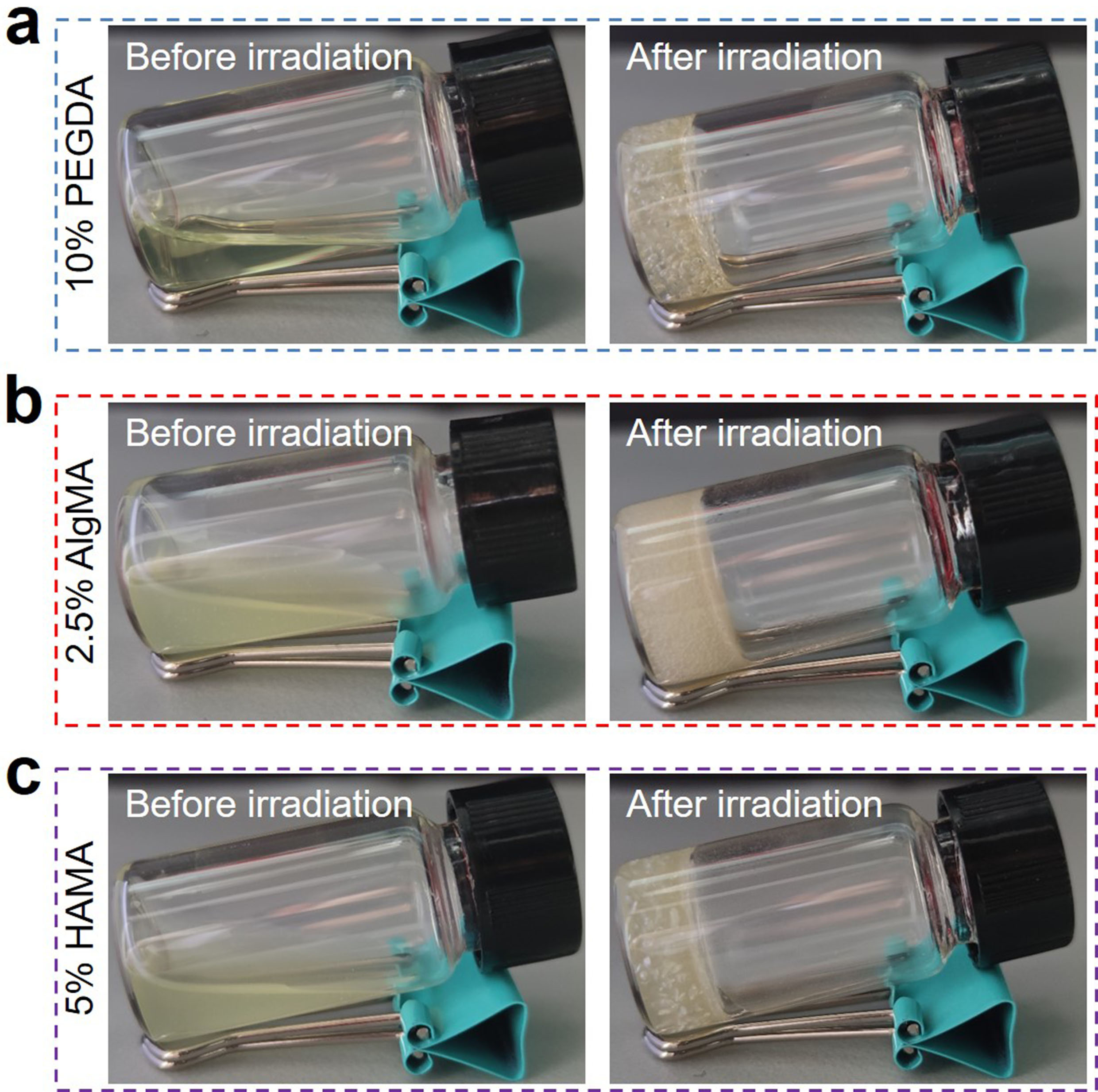


**Figure S7.** Photographs of (a) PEGDA, (b) AlgMA and (c) HAMA hydrogels with the concentrations of 10%, 2.5% and 5% respectively before and after UV light irradiation.


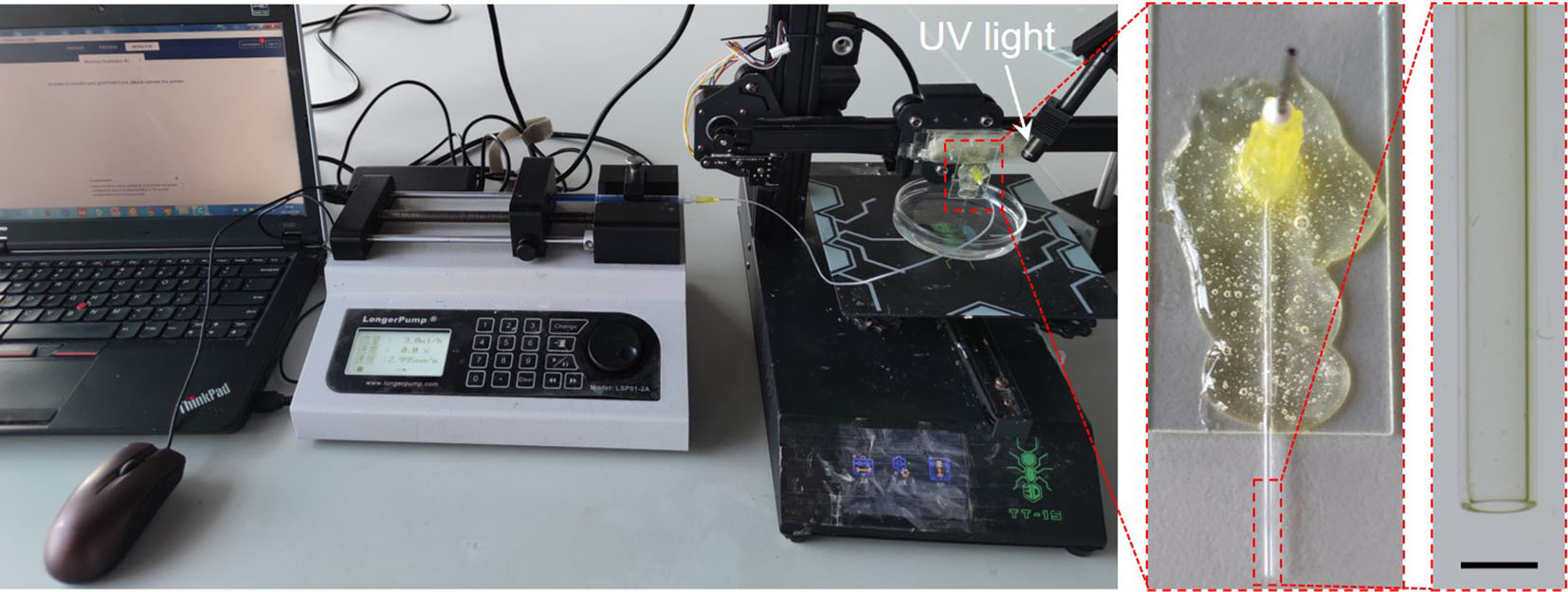


**Figure S8.** Photograph of the microfluidic 3D printer with capillary microfluidic chips for scaffold printing. Scale bar, 1000 μm.


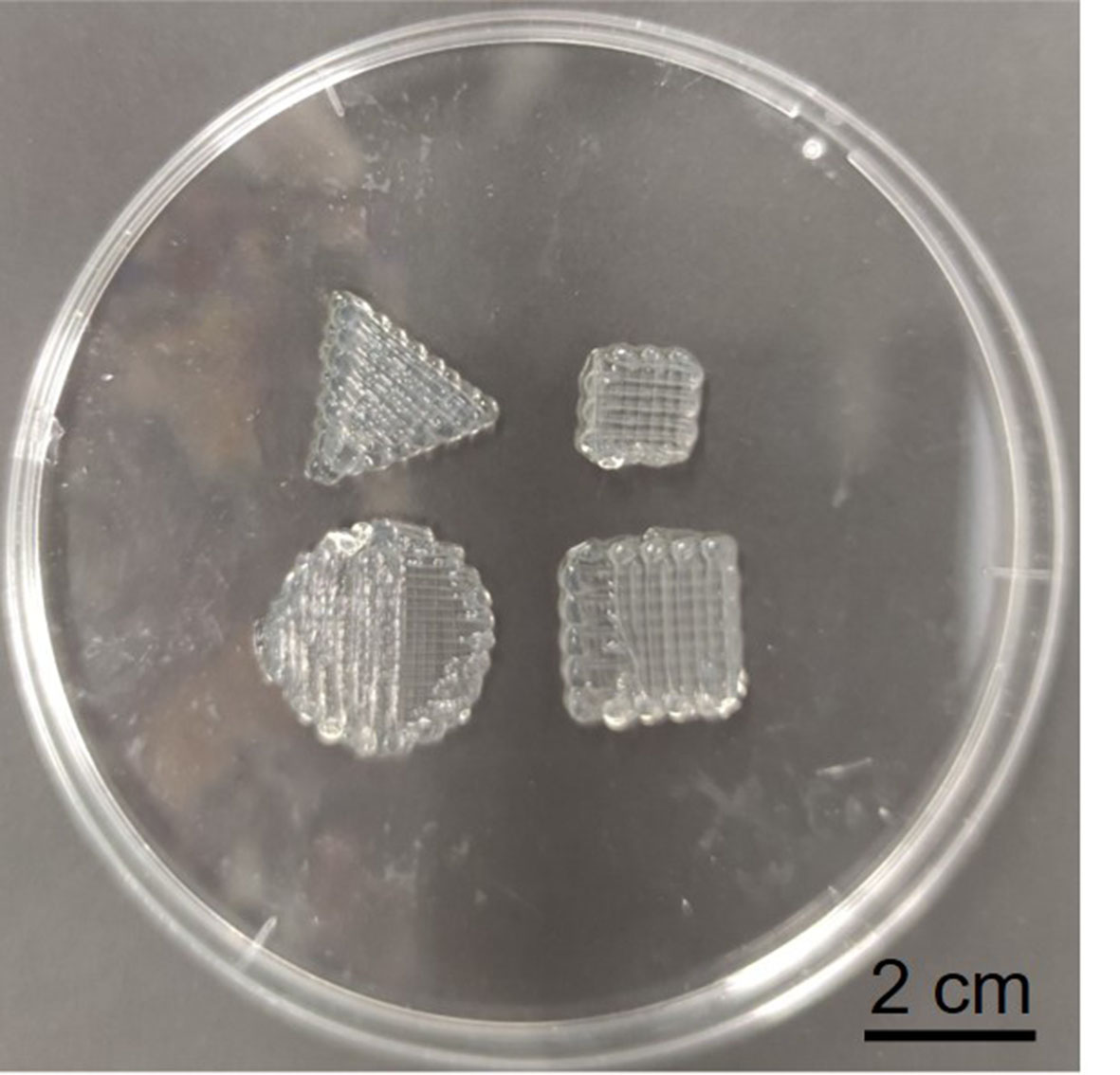


**Figure S9.** Photograph of representative 3D printing GelMA scaffolds with designed sizes and shapes.


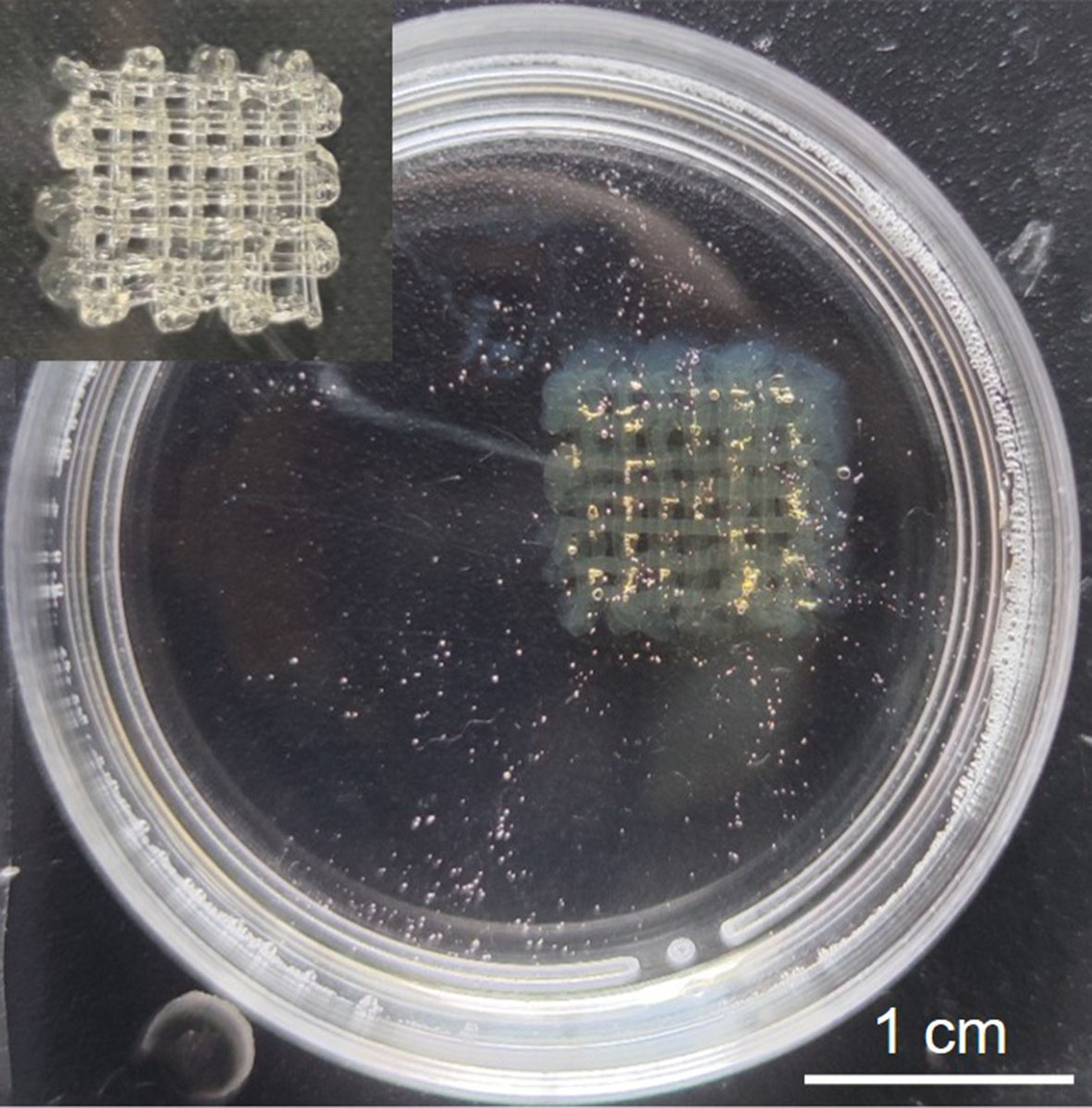


**Figure S10.** Photograph of the 3D printing Pt-GelMA scaffold.
